# In silico analysis of ventricular action potential with a current–voltage‐time representation: Thresholds, membrane resistance, repolarization reserve

**DOI:** 10.14814/phy2.70085

**Published:** 2024-11-12

**Authors:** Massimiliano Zaniboni

**Affiliations:** ^1^ Department of Chemistry, Life Sciences and Environmental Sustainability University of Parma (Italy) ‐ Parco Area Delle Scienze Parma Italy

**Keywords:** cardiac action potential, membrane resistance during the action potential, quasi‐instantaneous IV curves, reserve of repolarization, threshold for all‐or‐none‐repolarization, threshold for excitation, ventricular repolarization

## Abstract

The waveform of ventricular action potential (AP) is a key determinant of the cardiac cycle, a marker of beating pathophysiology, and a target for anti‐arrhythmic drug design. The information contained in the waveform, though, is limited to the actual dynamics of the AP under consideration. By measuring quasi‐instantaneous current–voltage relationships during repolarization, I propose a three‐dimensional representation of the ventricular AP which includes potential dynamic responses that the beat can show when electrically perturbed. This representation is described in the case of a numerically reconstructed ventricular AP, but it can be, at least partially, derived in real cardiomyocytes. Simulation allows to disclose the potentialities and the limitations of the approach, that can be extended to any non‐cardiac AP. By reporting, at any AP time, the ion current available within the physiological membrane potential range at that time, the representation makes all together available: (1) refractory period, (2) thresholds for eliciting full or calcium‐driven APs, (3) threshold for all‐or‐none repolarization, (4) membrane resistance during repolarization, (5) the safety of membrane repolarization. It provides further evidence of a negative membrane resistance during the late phase of ventricular AP and a quantitative estimate of repolarization reserve (RR), key determinants of repolarization dynamics.

## 1 INTRODUCTION

The way we measure and represent physical events frequently conditions the way we understand their dynamics and design new tools to control them. In the case of membrane excitability, the phenomenon was first measured as an action current (Bernstein, 1868; Du Bois‐Reymond, 1848) and only later the measure of membrane potential (*V*_m_) displacement during excitation, the action potential (AP), became the elected tool in electrophysiological studies. The intermingled nature of membrane currents and *V*_m_ became clear with the numerical reconstruction of the AP based on the kinetics of ion currents, which was formulated by Hodgkin and Huxley (Hodgkin & Huxley, 1952) (HH) and gave rise, together with a deeper understanding of cellular physiology, to the new field of computational electrophysiology (Koch, 1998). The four ordinary differential equations of the HH model for the squid giant axon were soon implemented with additional details to describe different preparations, eventually leading to the formulation of HH‐type cardiac AP models (Noble, 1960, 1962a, 1962b, 2007), which kept growing in complexity over the years (Noble et al., 2012). Numerical models of human cardiac APs are used to study physiological mechanisms, pharmacological regulation, and pathophysiological alterations of cellular functions, and are now an essential tool in cardiac electrophysiology at the cellular, tissue, and whole organ level. Quasi‐instantaneous current voltage relations during the course of the AP have first been measured by Hodgkin and Huxley (Hodgkin & Huxley, 1952) in the giant axon of Loligo, and then adopted, among few others, by Noble and Tsien in cardiac Purkinje fibers (Noble & Tsien, 1969a), by Beeler and Reuter in the way to set up their AP model of cardiac ventricular AP (Beeler & Reuter, 1977), and by Goldman and Morad in frog ventricular cells (Goldman & Morad, 1977). My colleagues and I have measured quasi‐instantaneous current–voltage relations in patch clamped isolated guinea pig ventricular myocytes (Zaniboni et al., 2000) at selected times during AP repolarization, in order to estimate membrane resistance (*R*_m_) at those times. In a following computational study we reported measurements of quasi‐instantaneous total membrane current *I*_m_‐voltage relations, where *I*_m_ = *I*_C_ + *I*_ion_, with *I*_C_ and *I*_ion_ the capacitive and ion current respectively (Zaniboni, 2011; Zaniboni et al., 2010). When these isochronal *I*_m_‐V relations were time‐aligned, the resulting surface intersected, by definition, the *I*_m_ = 0 plane along the AP trajectory and made it possible to unveil critical differences in ventricular APs displaying identical, or very similar, AP waveforms. By means of the same approach I showed, in two additional computational studies (Zaniboni, 2012a, 2012b) that the last phase of repolarization is self‐regenerative in sino‐atrial, atrial, Purkinje and ventricular APs. The difference introduced in the present study is in time aligning *I*_ion_‐*V*_m_, rather than *I*_m_‐*V*_m_, curves, thus capturing simultaneously in the same reconstructed surface, the *I*_ion_ flowing at each time during the AP waveform and its potential changes at different *V*_m_s.

Thus, by means of one of the most affirmed models of the human cardiac ventricular AP (O'Hara et al., 2011, Ord model) I introduce here a three‐dimensional AP representation showing the time‐evolution of quasi‐instantaneous IV curves during repolarization, IVT‐surface in short. The term quasi‐instantaneous, adopted in isolated cardiomyocytes studies (Hume et al., 1986), was due to the limited speed of voltage clamp control of *V*_m_. Numerical simulations do not suffer from this limitation, though, as I will show, the term remains, since an arbitrary time should be set for the ion currents gating to be manifest in the characteristic N‐shape profile of IV curves that give rise to the IVT‐surface.

I show that IVT‐surfaces provide a direct and intuitive measure of relevant dynamic features of membrane excitability that usually require separate and specific procedures to be accessed.

1. The intersection of the surfaces with the zero‐current plane identifies voltage thresholds for full excitation, for calcium current‐driven excitation, and for all‐or‐none repolarization (AONR).
2. It also unveils a narrow time window during late repolarization, where bi‐stable responses to cathodal stimulation in the form of calcium‐ or sodium‐driven APs can take place.
3. IVT‐surfaces allow to identify *V*_m_ values during AP plateau that, when reached, lead to self‐regenerative repolarization (SRR).
4. They also unveil a time window during AP trajectory when repolarization is already self‐regenerating.
5. The (I,V,T)‐states of the AP belong to the IVT surface and provide, when projected onto the orthogonal planes of the (I,V,T)‐space, in turn, the AP profile, its current–voltage phase plot, and the time course of *I*_ion_ during AP.
6. IVT‐surfaces make readily accessible the measure of membrane resistance (*R*_m_) during AP repolarization and, with that, a time window when *R*_m_ assumes negative values.
7. Finally, and most importantly, they show, at any repolarization time, the amount of charge available to cross the membrane and guarantee or not the safety of repolarization. I show that they can be used to quantify reserve of repolarization (RR) of the ventricular AP.

The natural development of the present investigation is to extend the 3D representation of the AP also to propagation, first for example in cell pairs, and then in mono‐, bi‐, and tri‐dimensional cell arrangements. These aspects, though, cannot possibly fit the present study, which is uniquely focused on the physiology of the non‐propagated, that is, single cell, ventricular AP.

As defined in this study, IVT‐surfaces can be applied to any numerical AP model, can in principle be measured, though with limitations that I will discuss, in single isolated cardiac myocytes, and provide a new way to look at the AP dynamics, particularly for screening overall effects of complex pathological events or pharmacological interventions involving transitions from normal to abnormal repolarization.

## 2 METHODS

The simulations presented in this study have been performed with the O'Hara et al. AP model (O'Hara et al., 2011, ORd model in short), which is one of the most adopted formulations to reproduce human ventricular electrical activity. The stiff ‘ode15s’ solver built into the R2023a version of Matlab (TheMath‐Works, Inc., USA) was used to integrate model's equations. All simulations were run on a PC with Intel(R) Core (TM) i7, 2.8GHz CPU. APs were elicited by simulating 0.5 ms‐long current injections with an amplitude 50% above current threshold. AP duration was measured as the time between the maximum first derivative of membrane potential (*V*_m_) during the initial fast depolarization phase and the time during repolarization when *V*_m_ reached the value of −60 mV. Initial conditions at different basic cycle lengths (BCLs), or for any modulation of maximum conductance of ion channels or of ion concentrations, were measured after conditioning pacing of 1000 beats. The BCL = 800 ms was adopted in most of the simulations as it is close to the physiological beating cycle length (CL) of the human ventricle. Supplementary videos were prepared by mounting individual simulation plots with a custom‐made Matlab routine.

## 3 RESULTS

### 3.1 Instantaneous IV curves and the IVT‐surface

**FIGURE 1:**
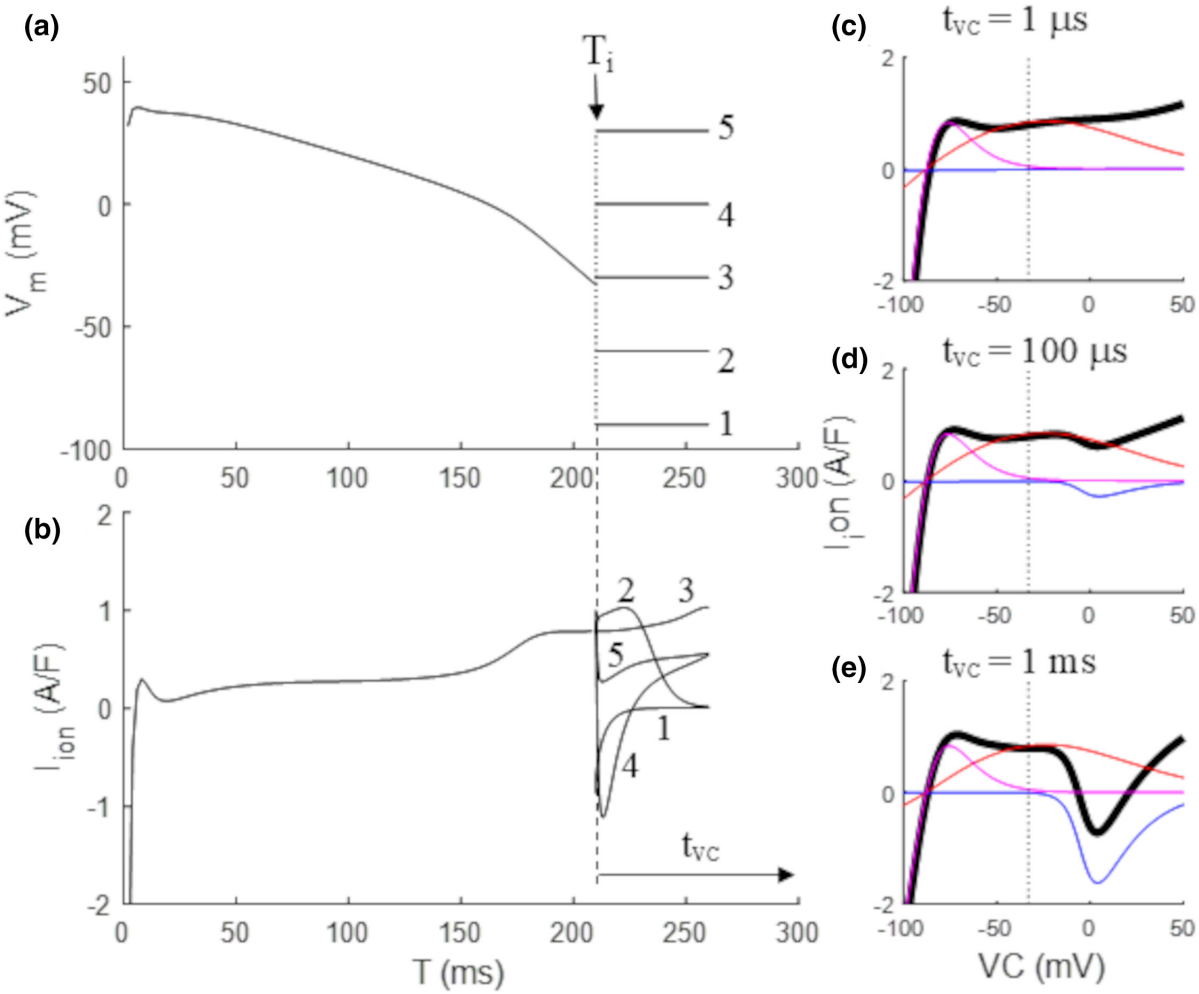
Experimental protocol for measuring quasi‐instantaneous IV curves. (a) During the repolarization phase of an ORd‐AP, at selected times (T = 210 ms in figure) the membrane potential is clamped for a time *t*_vc_, at values between −100 up to +50 mV (step 25 mV in figure, and step 2 mV in the rest of simulations of the study). (b) The *I*_ion_ current flowing before and during *t*_vc_ is reported. (c–e) IV_tvc_ curves for total *I*_ion_ (black) and for *I*_Kr_ (red), *I*_K1_ (magenta), and *I*_CaL_ are reported as measured for *t*_vc_ = 1, 100, and 1000 μs.

I first applied on the ORd model a previously described (Zaniboni, 2011, 2012a, 2012b; Zaniboni et al., 2010) protocol for measuring quasi‐instantaneous IV curves during the AP. Briefly, at different times T (step 2 ms in all the simulations) during AP repolarization, the *V*_m_ of the ORd equation system is clamped at different values (step 25 mV in Figure 1, step 2 mV in the rest of the study) and the corresponding total ion current (*I*_ion_) measured at a given time (*t*_vc_) during each clamp pulse (Figure 1a,b).

**FIGURE 2:**
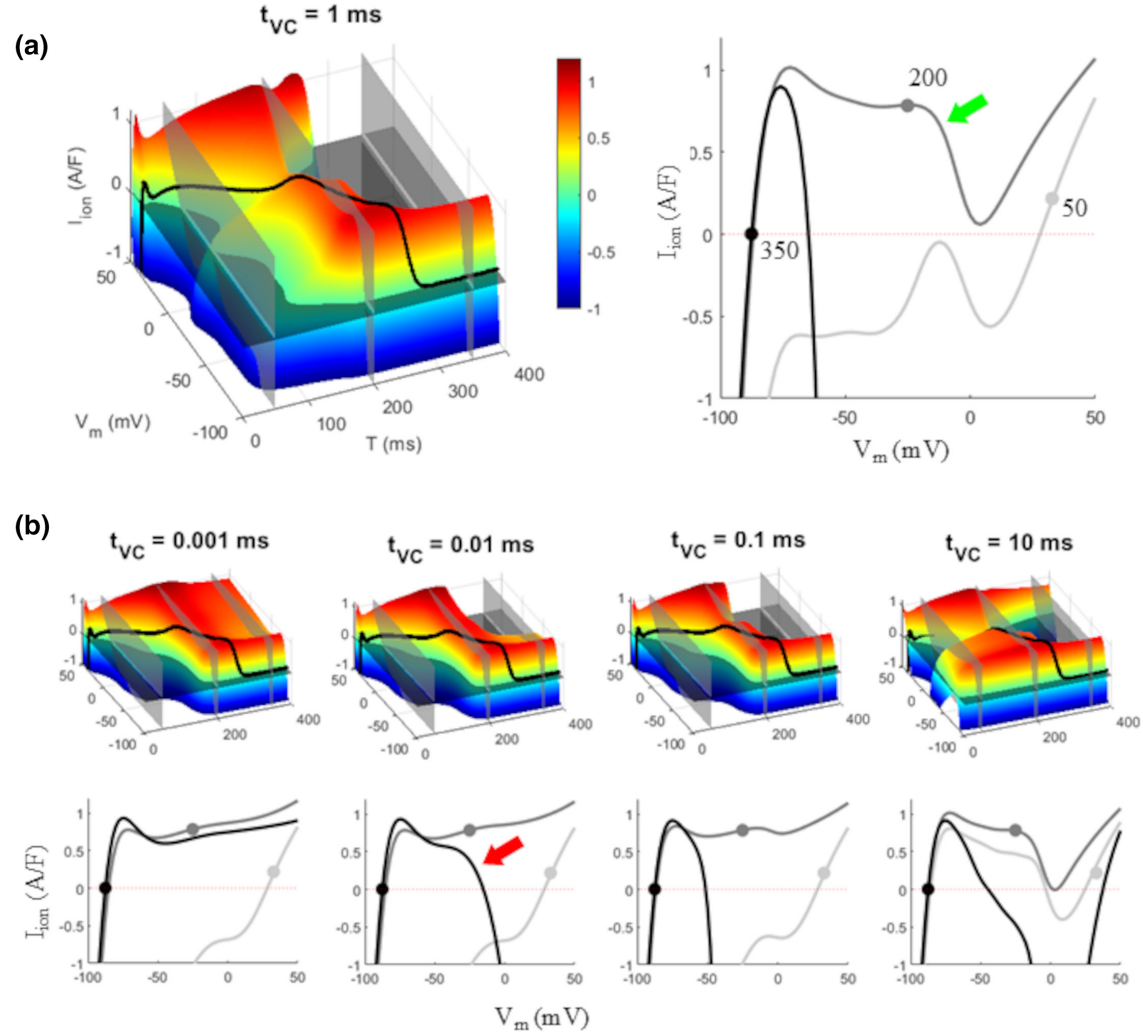
IVT‐surfaces at different *t*_vc_s. IV curves were measured as explained in Figure 1 at different *t*_vc_s and aligned along the T‐axis to reconstruct the IVT surfaces (step *V*_m_ = 2 mV, step T = 2 ms) in figure, for an AP conditioned at CL = 800 ms. (a, left) The IVT surface measured with *t*_vc_ = 1 ms with (a, right) selected IV curves taken at the times T indicated by transparent gray IV planes in the left panel. (b) IVT surfaces measured for different *t*_vc_s with the corresponding IV curves taken at the same times T. IV curves taken at times T = 50, 200, and 350 ms are reported in figure with light gray, dark gray, and black respectively. The dot on each curve represents the (I,V)‐state of the AP at the corresponding time T.

**FIGURE 3:**
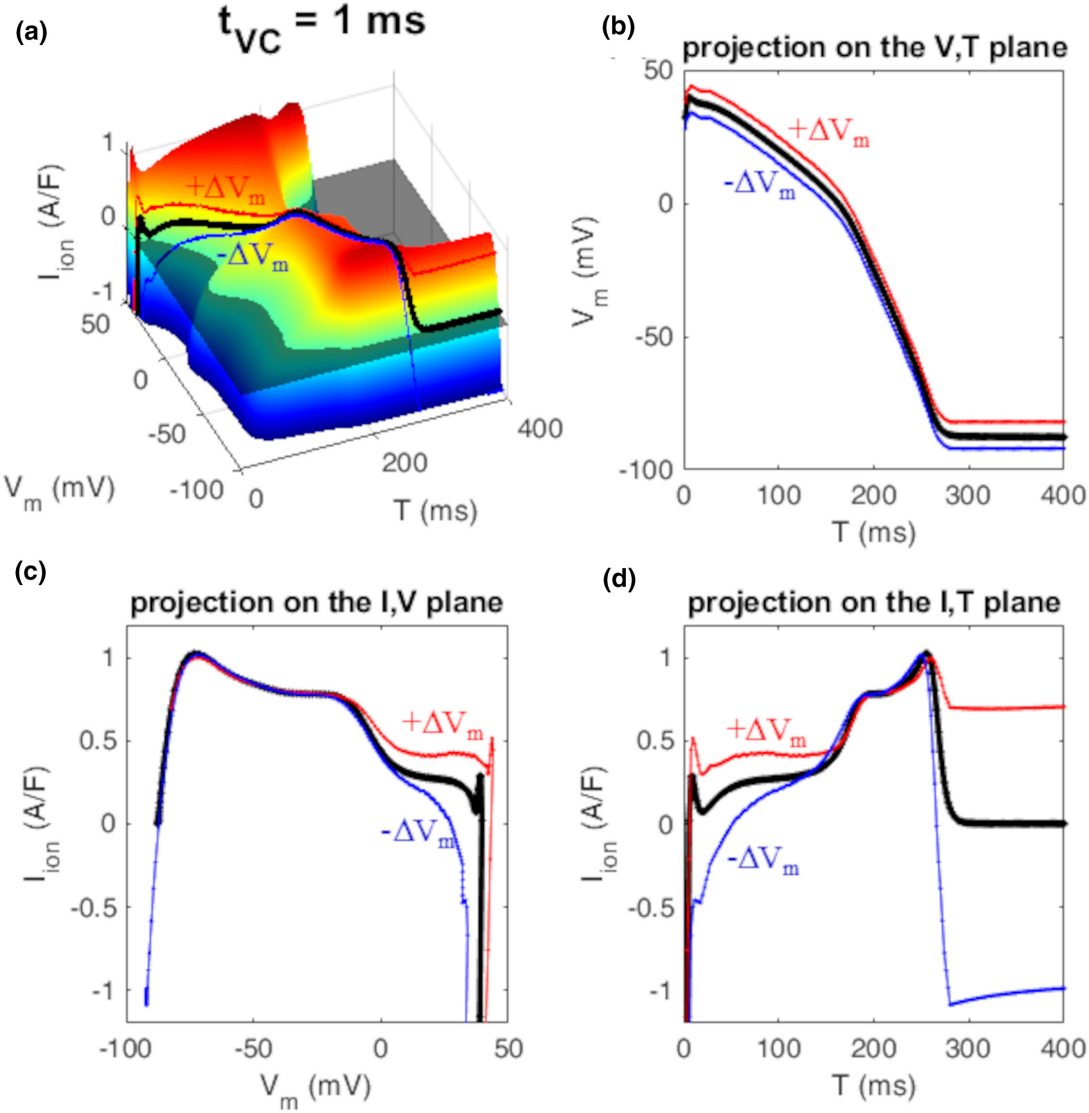
Projections on the 3 orthogonal planes. The projection of the 3D AP curve (bold black line) of the IVT surface (a) on the V,T plane provides the AP trajectory (b), on the I,V plane the phase‐plot (c), and on the I,T plane the time course of *I*_ion_ flowing during the AP repolarization (d). Red and blue curves in panels a, c, and d represent the way *I*_ion_ is modified when *V*_m_ of the AP, at each time T, is quasi‐instantly (1 ms) depolarized or hyperpolarized by 10 mV respectively (b).

All ion currents included in the ORd formulation are also measured at the same *t*_vc_. By reporting the values of *I*_ion_ flowing at time *t*_vc_ for each clamped *V*_m_, the quasi‐instantaneous IV_*t*vc_ (e.g. IV_0.01_ is that measured at 10 μs) relationship is obtained. In real membrane experiments *t*_vc_ cannot be smaller than 2–5 ms, due to the limitation of capacitive current compensation of the voltage clamp amplifiers. In numerical simulations *I*_ion_ can be measured at any *t*_vc_ allowed by the accuracy of numerical integration. Thus, Figure 1c–e represents the IV_*t*vc_ curves measured at time T = 210 ms and for *t*_vc_ = 1 μs, 100 μs, and 1 ms (bold black line), together with those of the main ion currents involved in AP repolarization. To note, the purely voltage‐dependent component of *I*_Kr_ (red) and *I*_K1_ (magenta) rectification in the IV_0.001_ curves of panel c. Time‐dependent non‐linearities appear only at later *t*_vc_, for example, *I*_CaL_ *V*_m_‐dependence starts to develop only for *t*_vc_ > 100 μs (compare blue curves in panels c–e). By measuring and time‐aligning IV_1_ curves every 2 ms during the AP, a three‐dimensional IVT‐surface, like that reported in Figure 2a, was constructed. To note, in previous studies (Zaniboni, 2011, 2012a, 2012b; Zaniboni et al., 2010) I have made use of IVT‐surfaces, though reporting the membrane current *I*_m_ = *I*_ion_ + *I*_C_ (*I*_C_ being the capacitive current) rather than *I*_ion_ on the z‐axis, with the obvious result that the surface was cutting the zero‐*I*_m_ level along the AP waveform. Compared to that, the representation adopted here has the advantage of showing, at any time, also the *I*_ion_ flowing during the AP (see also Figure 3) and, as will be explained later, of easily identifying thresholds.

Examples of IVT‐surfaces as measured with *t*_vc_ = 1, 10, 100, and 10 ms are also reported in Figure 2b. The AP waveform, that is, the collection of its (I,V,T)‐states, belongs to each surface, where it is reported as a three‐dimensional black curve. The projection of this curve onto the three orthogonal planes of the (I,V,T)‐space provides, in turn, the AP waveform, its voltage–current phase plot, and the time course of *I*_ion_ during the AP (Figure 3). The way these three curves are modified by quasi‐instantaneous depo/hyper‐polarizations (ΔV_m_ = 10 mV) at each time T are represented in Figure 3b–d, and on the corresponding 3D surface (panel a). Whereas for *t*_vc_ <2–3 ms the 3D AP waveform is always clearly visible lying on the IVT‐surface (first 3 surfaces of Figure 2b), for larger *t*_vc_ values it falls, during the course of AP repolarization, beneath the surface, due to significant activation of time dependent ion currents (δ*I*_ion_) during *t*_vc_ (see right panel of Figure 2b at *t*_vc_ = 10 ms). In other words, since the 3D surface represents currents (*I*_ion_ + δ*I*_ion_) measured at T + *t*_vc_, when *t*_vc_ is small enough, δ*I*_ion_ → 0 and the (I,V,T)‐state of the AP shows the *I*_ion_ flowing during the AP trajectory. For longer *t*_vc_s (>2–3 ms), particularly during late plateau phase, δ*I*_ion_ is not negligible and the state of AP (*I*_ion_,V,T) falls occasionally below the measured surface (*I*_ion_ + δ*I*_ion_,V,T). For a clearer appreciation of the evolution of IVT‐surfaces with increasing *t*_vc_, see also Movie S1.

Isochronal slices of each surface of Figure 2, at times T = 50, 200, and 350 ms (transparent gray planes in figure), provide the three IV curves reported in right panel a and bottom panel b, where the dot on each curve represents the (I,V,T)‐state of the AP at that time. In the IVT‐surface of panel a, for example, at time T = 50 ms, the IV curve is the one reported on the right in light gray, with AP values of *V*_m_ and *I*_ion_ respectively 32.7 mV and 0.22 A/F. Surfaces are qualitatively very similar within a large *t*_vc_ window, particularly in the *V*_m_ range around the AP potential and until final repolarization. At later times (T >200 ms) large inward currents (first *I*_CaL_ and then *I*_Na_) activate very early (*t*_vc_ >10 μs) at potential values depolarized with respect to that of the AP. Green and red arrows in Figure 2 show the downward deflection of the IV curve due to activation of *I*_CaL_ (at time T = 200 ms, dark gray) and *I*_Na_ (at time T = 350 ms, black) respectively, the first, as expected, at more depolarized potentials, the second closer to resting *V*_m_. A good compromise to capture the initial activation dynamics of both currents seems to be *t*_vc_ = 1 ms (see also videos in Movies S1 and S2).

**FIGURE 4:**
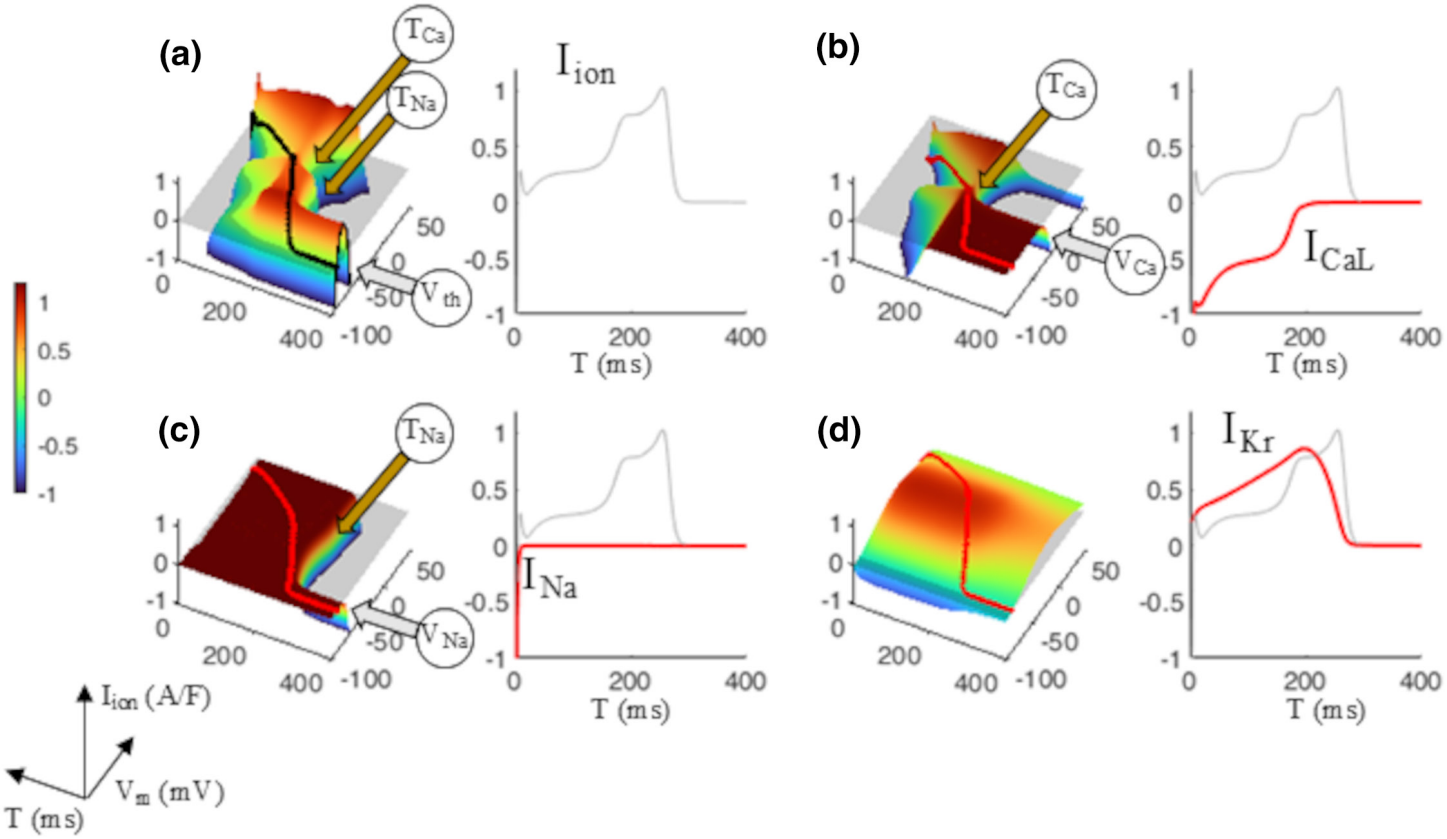
Ion currents contributions to the AP surface. The IVT_1ms_ surface is reported in left (a). The projection on the IT plane of the (I,V,T)‐states of the AP (black curve on the surface) represents the time course of *I*_ion_ during the AP and is reported on the right of the same panel, like in Figure 3d. IVT surfaces for *I*_CaL_ (b), *I*_Na_ (c), and *I*_Kr_, are also reported in figure. The projection of the (I,V,T)‐states of the AP (red curve on each surface) on the IT plane provides the time course of the corresponding currents during the AP. These are reported in the right side of panels b, c, and d in red, superimposed with the total ion current (gray).

The contribution of three ion currents of the ORd model to the IVT‐surface of Figure 2a is reported in Figure 4. The IVT‐surfaces for *I*_CaL_ (panel b), *I*_Na_ (panel c), and *I*_Kr_ (panel d) are derived with the same protocol reported in Figure 1 by only measuring the values of those currents instead of total *I*_ion_ during the voltage clamp steps. As we will see in the next paragraph, the *V*_m_ at which *I*_Na_ activates (*V*_Na_, gray arrow panel c) starting from *T*_Na_ (brown arrow) determines, together with the *I*_K1_ component, the voltage threshold (V_th_, panel a) for excitation at rest. The threshold for *I*_CaL_ activation appears earlier (*T*_Ca_, panels a and b) during AP repolarization and at more depolarized potential (*V*_Ca_) (panel b). The latter is responsible for calcium‐dependent APs that can be elicited during the late phase of repolarization when *I*_Na_ is still completely inactivated (see also Figure 9), or when slow depolarizations partially or completely inactivate *I*_Na_ during diastole.

### 3.2 Thresholds

Quasi‐instantaneous IV‐curves measured during the AP, as already noted in early literature (Huxley, 1959; Noble & Tsien, 1969b), evolve from a linear to a cubic, N‐shaped, form as *t*_vc_ increases. The cubic curve cuts the zero current axis either in one or in three points, each of which is an equilibrium point (state), stable in case positive IV slope, unstable (threshold) otherwise (Izhikevich, 2010; Jack et al., 1975; Murray, 1993). Moreover, unstable (I,V,T)‐states lead to self‐regenerative (all‐or‐none) depolarization if corresponding *I*_ion_ is negative, AONR if it is positive. Thus, the inspection of the IVT‐surface readily provides *V*_m_ thresholds available at each AP time.

#### 3.2.1 Threshold for excitation

**FIGURE 5:**
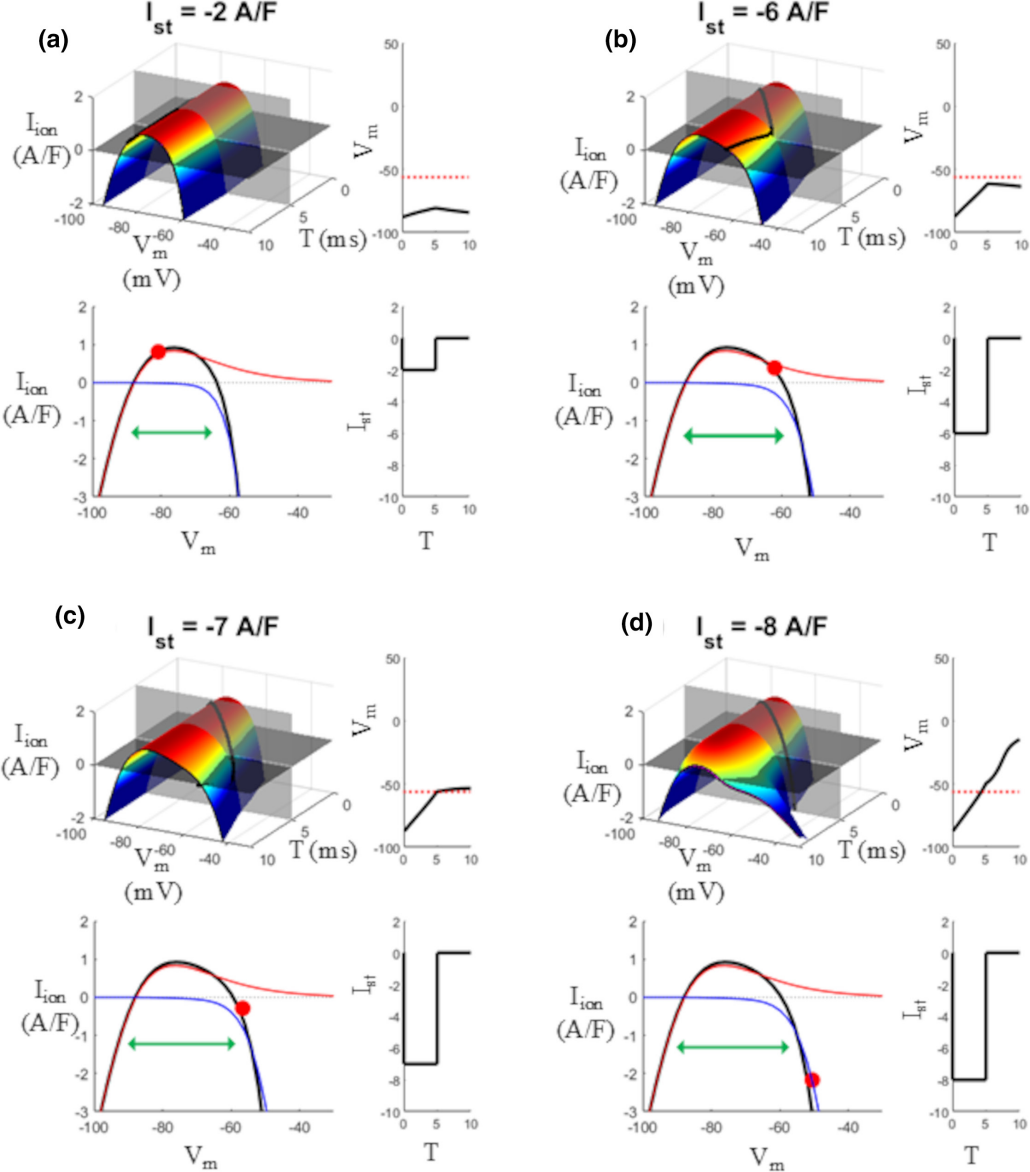
Voltage threshold for excitation. IVT_5ms_ surfaces were measured during simulations of sub‐threshold (a, b), threshold (c), and supra‐threshold (d) 5 ms depolarizing current injections. The (I,V,T)‐states of the membrane are reported, during the injections, on each surface as a black curve. The (I,V)‐state of the membrane at the end of current injection (T = 5 ms) is reported as a red dot on each IV curve. On the bottom of each panel, the IV curve taken at T = 5 ms is reported for total *I*_ion_ (black), *I*_Na_ (blue), and *I*_K1_ (red). The *V*_m_ threshold is given in bottom panel c by the *V*_m_ value (~ −58 mV) where the total IV curve (black) intersects the zero‐current level. On the right of each panel, current injections (bottom) and *V*_m_ displacements (top) are also reported.

When the AP waveform reaches the resting state, for example, for times T > ~300 ms in the simulation of Figure 2a, the surface assumes a nearly parabolic cylindrical shape (see Figures 2a, 3a, and 4a) whose intersections with the zero current plane describe the stable resting state at −87 mV and the unstable threshold for excitation at −58 mV. Figure 5 shows a magnified representation of this condition (*t*_vc_ = 5 ms), where the membrane at rest undergoes a subthreshold (A and B), threshold (C) and supra‐threshold (D) 5 ms constant current injection.

The (I,V,T)‐state of the membrane during injections is reported as a black curve on each surface. The projection of this curve onto the T,V plane provides, in each top right panel, the voltage deflection in response to the corresponding current stimulus (on the bottom). The contribution of *I*_K1_ (red) and *I*_Na_ (blue) to the total IV curve (black) is reported in bottom left corner of each panel, all taken at time T = 5 ms at the end of the current pulse (gray transparent vertical plane cutting each surface). The longer the membrane endures at depolarized sub‐threshold potentials or during the initial depolarization phase (these conditions increase from a to d) the less is the contribution of *I*_Na_, due to its partial inactivation, to the total *I*_ion_‐V‐curve that, as a consequence, will tend to resemble more the *I*_K1_‐V curve, which causes the widening observed in its shape (green double arrows, from a to d). The 3D surfaces show in this case, at each time T, and together with the *V*_m_ displacement, the amount of ion current that the injected stimulus has to counteract in order to reach the unstable equilibrium point of the threshold.

**FIGURE 6:**
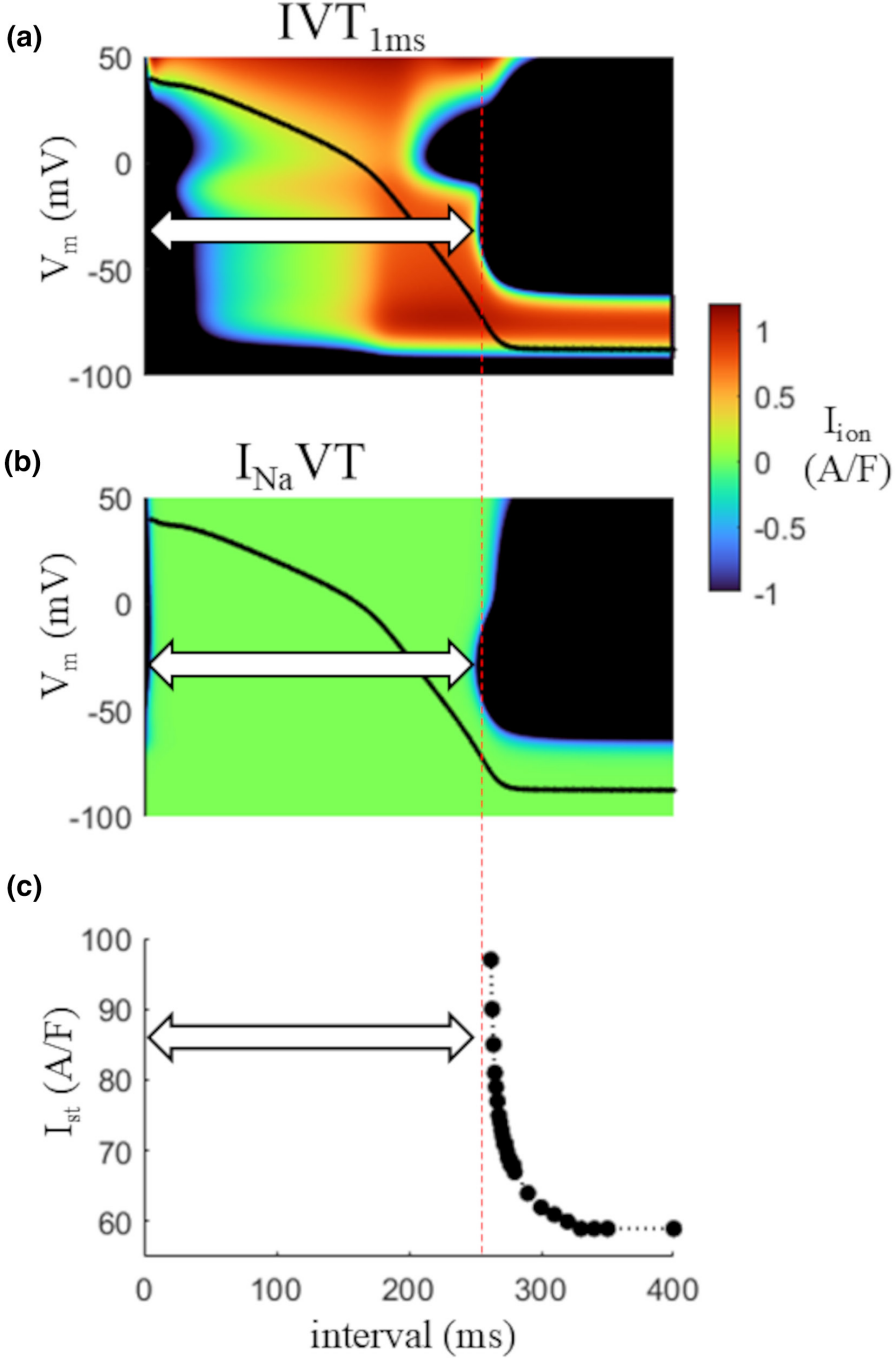
Absolute refractory period. Top view of the same IVT_1ms_ surface of Figure 4a for *I*_ion_ (a) and for *I*_Na_ (b). (c) Strength‐interval curve measured by injecting, right after an AP (same initial conditions as in a) and as the interval with respect to the preceding AP decreases, depolarizing current pulses of increasing amplitude. The liminal current corresponding to each interval is reported in figure. White double arrows in each panel show the absolute refractory time window.

The inspection of the IVT surface of Figure 4a from above (Figure 6a) allows appreciating the length of the absolute refractory period (double arrow), which coincides with the time window where no *I*_Na_ is available at any *V*_m_ (panel b, where the green color indicates *I*_Na_ = 0, unavailable at any *V*_m_). The same absolute refractory period was derived with conventional strength‐interval curve and reported in panel c for comparison.

#### 3.2.2 Transition to self‐regenerating repolarization (SRR)

**FIGURE 7:**
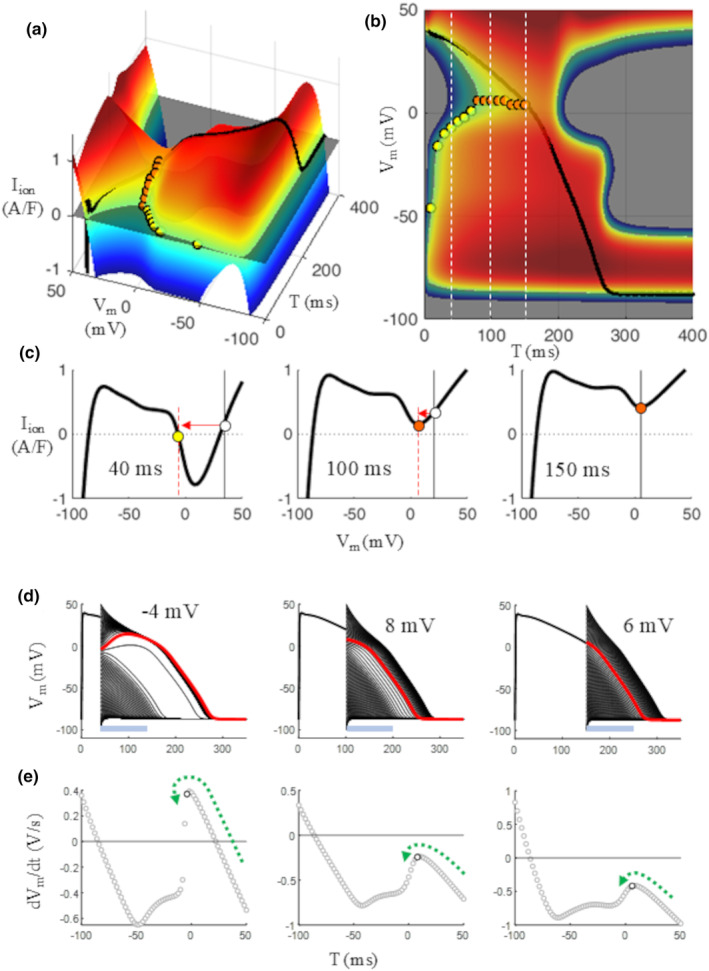
Voltage threshold for AONR. (a, b) Side and top view of the IVT_5ms_ surface. For T < 80 ms, threshold for AONR (yellow dot) is found where voltage displacement of the (I,V,T)‐state (withe dot) reaches the value where the negative sloping limb of the corresponding IV curve cuts the zero current level (left panel c). For 80 ≤ T ≤ 150 ms local minima of the corresponding IV curve (middle and right panels c) mark *V*_m_ values that, when reached and overcome by anodal stimuli, can make repolarization self‐regenerating. (d) Transition to SRR was also measured by voltage clamping *V*_m_ a different times T during plateau for 1 ms, and by measuring the average value of the *V*_m_ derivative in the first 100 ms after the clamp release (light blue bars). The *V*_m_ of the transition was taken as the first value (highlighted white dots in panels e) when the derivative start to decrease abruptly after a linear increase (green arrows).

IVT‐surfaces can readily reveal thresholds for AONR during the AP at the intersection between the surface and the zero‐current plane (yellow dots in Figure 7a–c). According to dynamic system theory, a threshold for AONR exists, strictly speaking, only for T < 80 ms. In this time window, an anodal current of adequate intensity can displace *V*_m_ to threshold (red arrow, left panel c). For 80 ≤ T ≤ 150 ms there is no real threshold but still an anodal stimulation can bring *V*_m_ to exceed the local minimum of the IV curve and reach its negative sloping limb, thus leading to SRR (middle and right panels of Figure 7c, orange dots). These local minima mark, in other words, a transition between the tendency of the membrane to rapidly recover the resting potential and the tendency to do it along the unperturbed AP trajectory. Local minima of this type (orange dots) mark an AP time window where SRR can be reached without a clear difference between the “all” and the “none” behavior.

**FIGURE 8:**
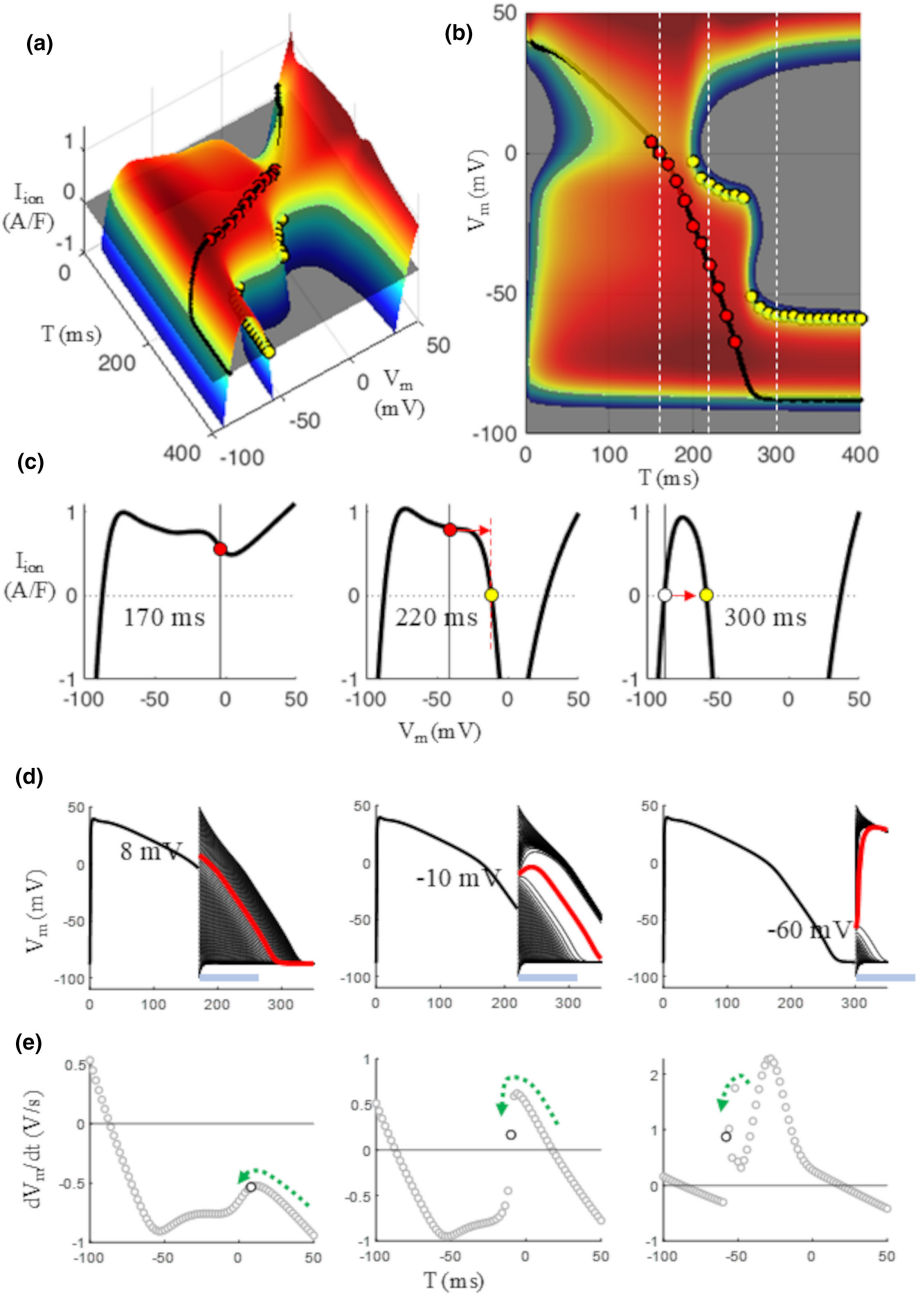
Voltage thresholds during late repolarization. The presence of thresholds is investigated at later times during AP. For 150 ≤ T ≤ 250 ms the (I,V,T)‐states of the membrane are already on a negative sloping limb of the corresponding IV curves (red dots in panels a–c), and repolarization is therefore self‐regenerative. In the first half of this window, no other thresholds are present. In the second half, for 200 ≤ T ≤ 260 ms, a threshold for *I*_CaL_‐driven AP is available in depolarizing direction when the curve crosses the zero‐current axis. For T ≥ 270 ms the (I,V,T)‐state of the membrane is at its rest (white dot on right panel c) and a threshold for *I*_Na_‐driven AP is available in depolarizing direction. Explanation of panels d and e like in Figure 7.

Though the difference is not clear, a qualitative difference does exist, and a qualitative transition to SRR for T ≤ 150 ms, either through a threshold or not, is in fact made manifest by means of a protocol previously described (Beeler & Reuter, 1977) and applied here on the ORd model (Figure 7d,e). During a simulated AP and for increasing times T, *V*_m_ is clamped for *t*_vc_ = 1 ms at values from +50 to −100 mV. At the release of the clamp, and for times T < 80 ms, a clear threshold for AONR is evident (Figure 7d). To make threshold discrimination less arbitrary, I measured the first time derivative of each *V*_m_ trace in the 100 ms after the clamp and reported its average as a white dot in panel e. The 100 ms window was chosen as it approximately amounts the maximum self‐regenerating repolarization time (see light blue horizontal bars in panels d), in order to compare corresponding *V*_m_ trajectories to those not showing SRR. Slightly shorter or longer time choices do not change the results qualitatively nor the *V*_m_ values at which transition is found to occur. When a threshold is present (T < 80 ms) there is a sharp transition in the first derivative of repolarization, starting from depolarized potentials and moving towards polarization (green arrows in Figure 7e). When, for 80 ≤ T ≤ 150 mV, SRR is reached but not through a definite threshold, a qualitative transition can still be measured (middle and right panel e). As repolarization proceeds (Figure 8), for 150 ≤ T ≤ 250 ms, the (I,V,T)‐state of the membrane during AP is already on the negative limb of the corresponding IV curve, thus AP repolarization is already self‐regenerating (left panel d). Corresponding (I,V,T)‐AP‐states are reported as red dots in Figure 8a–c.

#### 3.2.3 Threshold for calcium‐ and sodium‐AP

For 200 ≤ T ≤ 260 ms the threshold for an *I*_CaL_ – driven AP is clearly visible at the intersection between the IVT‐surface and the zero‐current plane (Figure 8a,b, yellow dots) and in the middle panels c–e. For T ≥ 270 ms, *I*_Na_ activation, when reached fast enough (see later discussion), overwhelms that of *I*_CaL_ and, within few tens of milliseconds, the resting excitability threshold described in Figure 5 is recovered.

**FIGURE 9:**
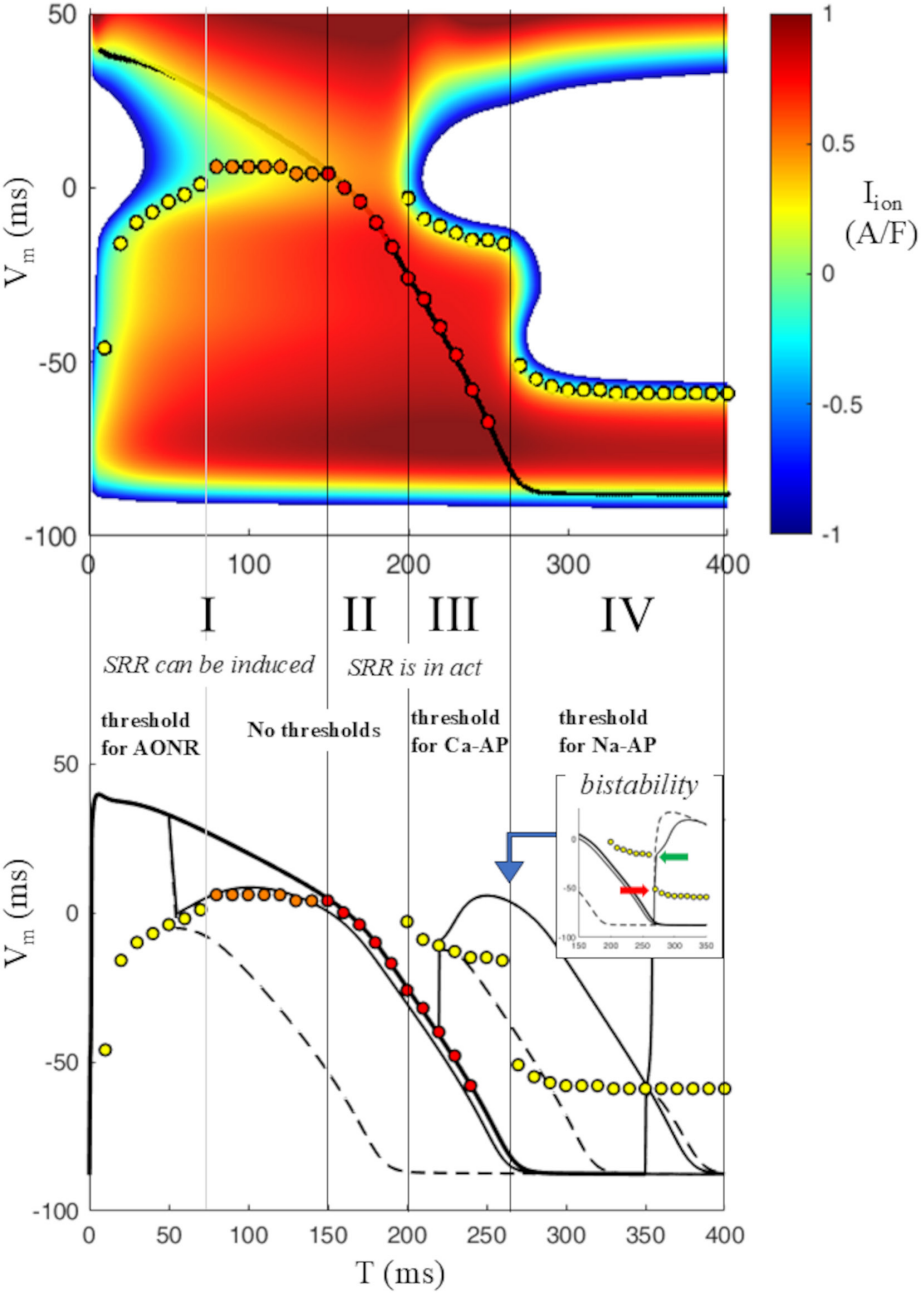
Summary of thresholds. Top panel: A summary of thresholds identified on the IVT_5ms_‐surface is reported. Region I identifies a time window when SRR can be induce, either through a threshold (yellow dots) or only by reaching the negative‐sloping limb of isochronal IV curves (orange dots). No *V*_m_ thresholds are available in region II, where (I,V,T)‐states (red dots) belong to the negative‐sloping limb of IV curves and repolarization is self‐regenerating. Voltage threshold for *I*_CaL_ activation is available during region III at depolarized potentials. Cathodal current injections at this time can bring *V*_m_ to this state and elicit *I*_CaL_‐driven APs. The voltage threshold for *I*_Na_ activation is available in region IV at depolarized potentials. Bottom panel: Sub‐threshold voltage displacements are reported as broken lines, supra‐threshold as continuous lines, for anodal (region I) and cathodal (regions III and IV) current injections. The inset of bottom panel indicates (blue arrow) the narrow time window (between 260 and 270 ms) when bi‐stability, between calcium‐ or sodium‐driven APs can take place.

In order to schematize the information provided by IVT‐surfaces concerning thresholds and repolarization dynamics, I divide AP repolarization into four regions summarized in Figure 9. I will call region I that, for T < 150 ms, when SRR can be reached, either through a threshold (yellow dots, T < 80 ms) or not (orange dots, 80 ms ≤ T < 150 ms). Similarly, I will call region II (150 ≤ T < 200 ms) that when no thresholds are available. AP repolarization is self‐regenerating during region II (red dots along the AP trajectory). The time window for 200 ≤ T ≤ 260 ms will be called region III and is characterized by the availability of *I*_CaL_ threshold (yellow dots). For most of region III (T ≤ 256 ms) AP repolarization is also self‐regenerating (red dots). Finally, the time window for T ≥ 270 ms, when *I*_Na_ threshold can be reached, will be called region IV. *V*_m_ responses to sub‐threshold and supra‐threshold anodal (region I) and cathodal (regions III and IV) 0.5 ms current injections are also reported in the lower panel of the same figure. The inset of bottom panel of Figure 9 shows a further remarkable feature of late AP repolarization concerning the border between region III and IV. As the shape of the IVT‐surface suggests, there is a narrow time window, for 260 ≤ T ≤ 270 (blue arrow), characterized by bi‐stability, that is, both threshold for calcium‐ and sodium‐driven APs (green and red arrows respectively) can be reached, depending on the strength and for the same duration of the stimulus. *V*_m_ response to a 0.5 ms‐long cathodal current during this time window, elicits in fact a sodium‐driven AP (broken line) when the strength is −80 A/F, and a calcium‐driven AP (solid line) with a strength = −72 A/F.

### 3.3 Membrane resistance during the AP

**FIGURE 10:**
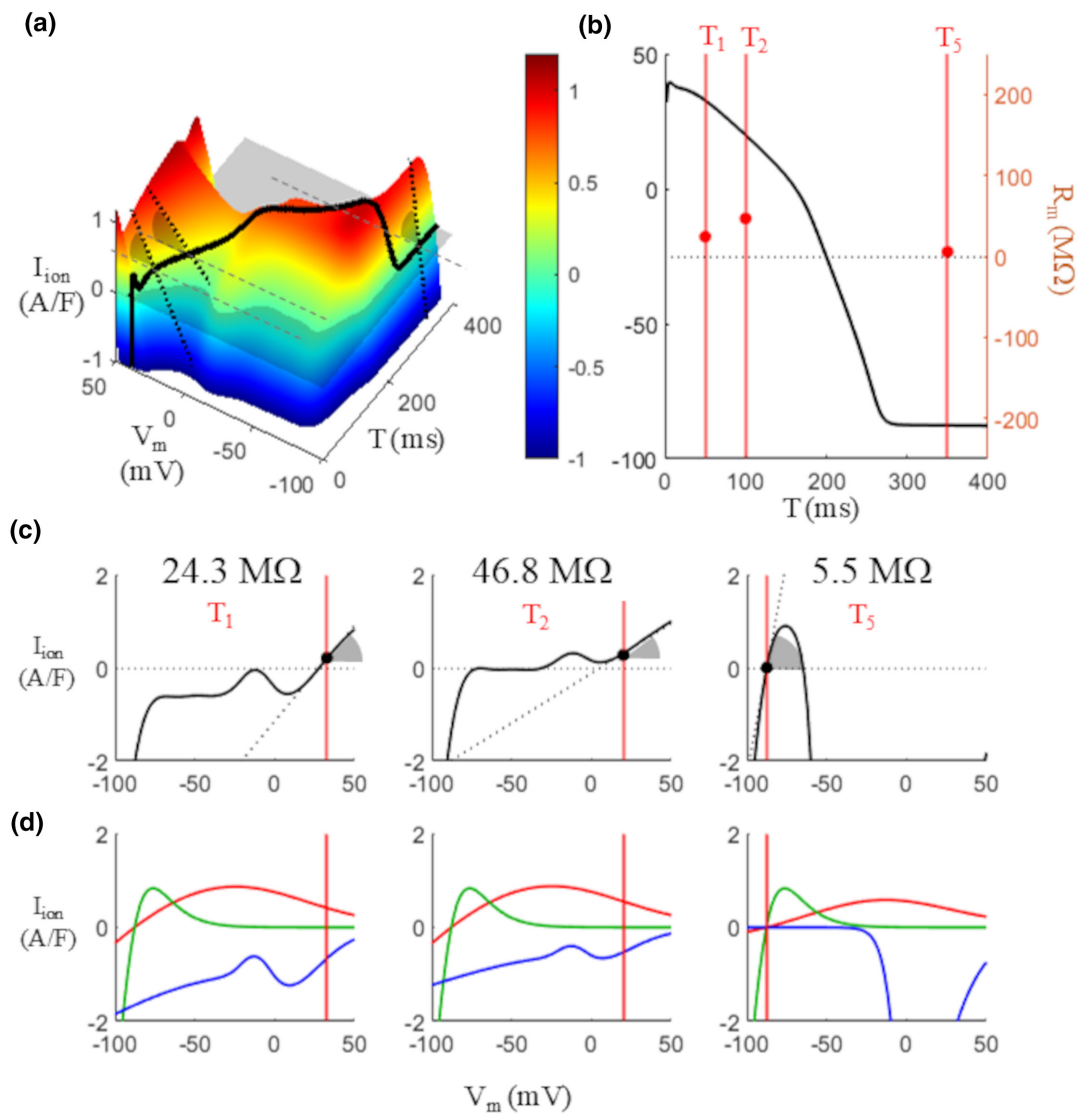
Membrane resistance during the AP: Positive values. (a) *R*_m_ values during AP are measured by taking the reciprocal of the angle of the IVT_1ms_ surface along IV planes (gray in figure). (b) Vertical lines represent the times T_1_, T_2_, and T_5_ (50, 100, and 350 ms respectively) when *R*_m_ was measured, and the red dots their values in MΩ (right axis). (c) IV curves at times T_1_, T_2_, and T_5_. Corresponding *R*_m_ value reported in each panel. (d) Corresponding IV curves for *I*_Kr_ (red), *I*_K1_ (green), and *I*_CaL_ (blue) at the 3 T values considered.

A further advantage of the IVT‐surface is the direct access to the measure of *R*_m_ during the AP trajectory. This can be estimated by taking the slope (in siemens) of the surface at any time T along the IV plane (gray shadowed angles in Figure 10a,c) and considering its reverse value (in Ohms).

**FIGURE 11:**
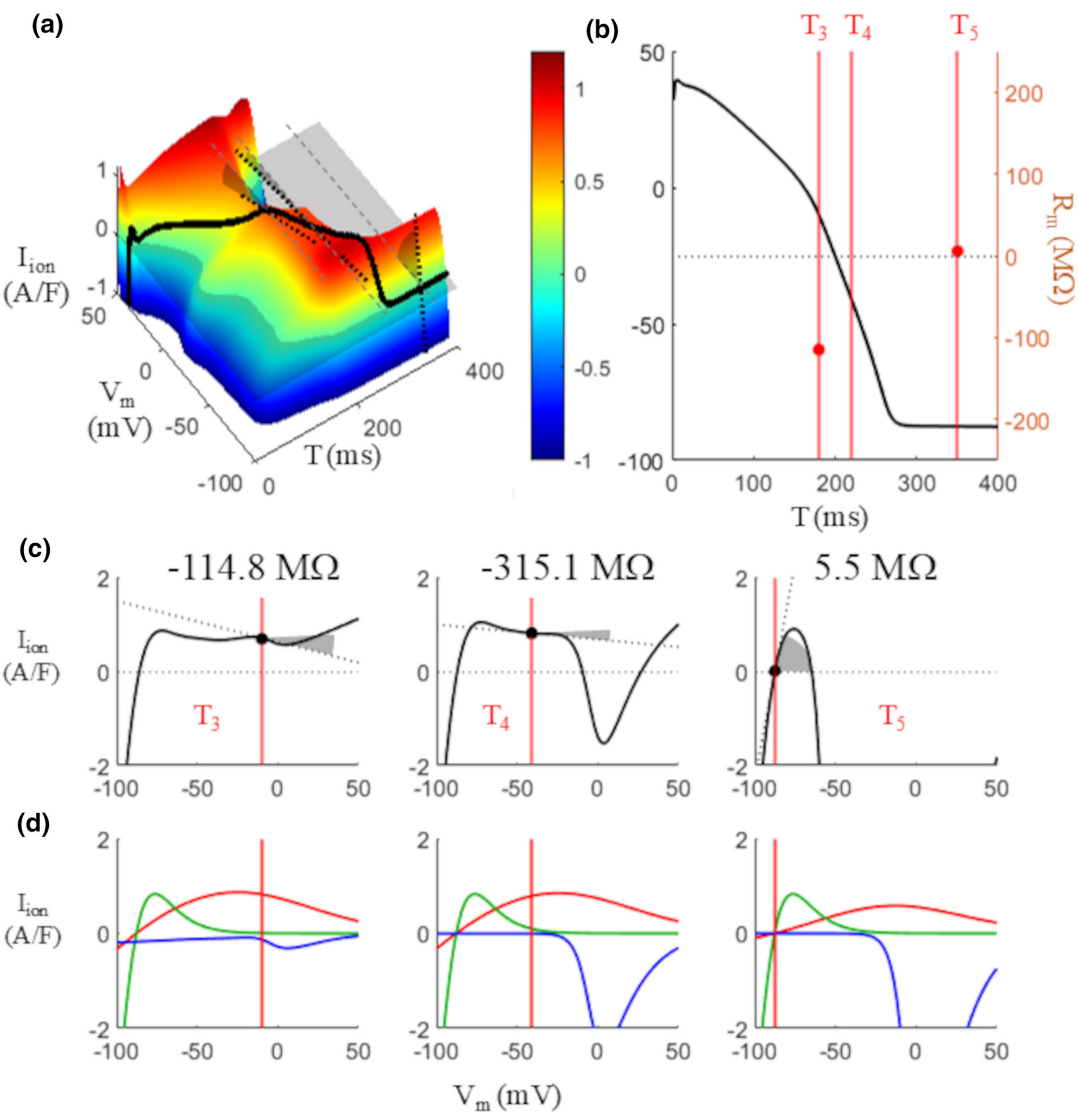
Membrane resistance during the AP: Negative values. Same as Figure 10; the diastolic value at T_5_ is the same for comparison. At times T_3_ = 180 and T_4_ = 220 ms, the slope of the surface along IV planes (gray shaded angles in panel a), and therefore that of the corresponding IV curves (panel c), is negative, and so are the *R*_m_ values measured at these times. (d) Corresponding IV curves for *I*_Kr_ (red), *I*_K1_ (green), and *I*_CaL_ (blue) at the 3 T values considered.

**FIGURE 12:**
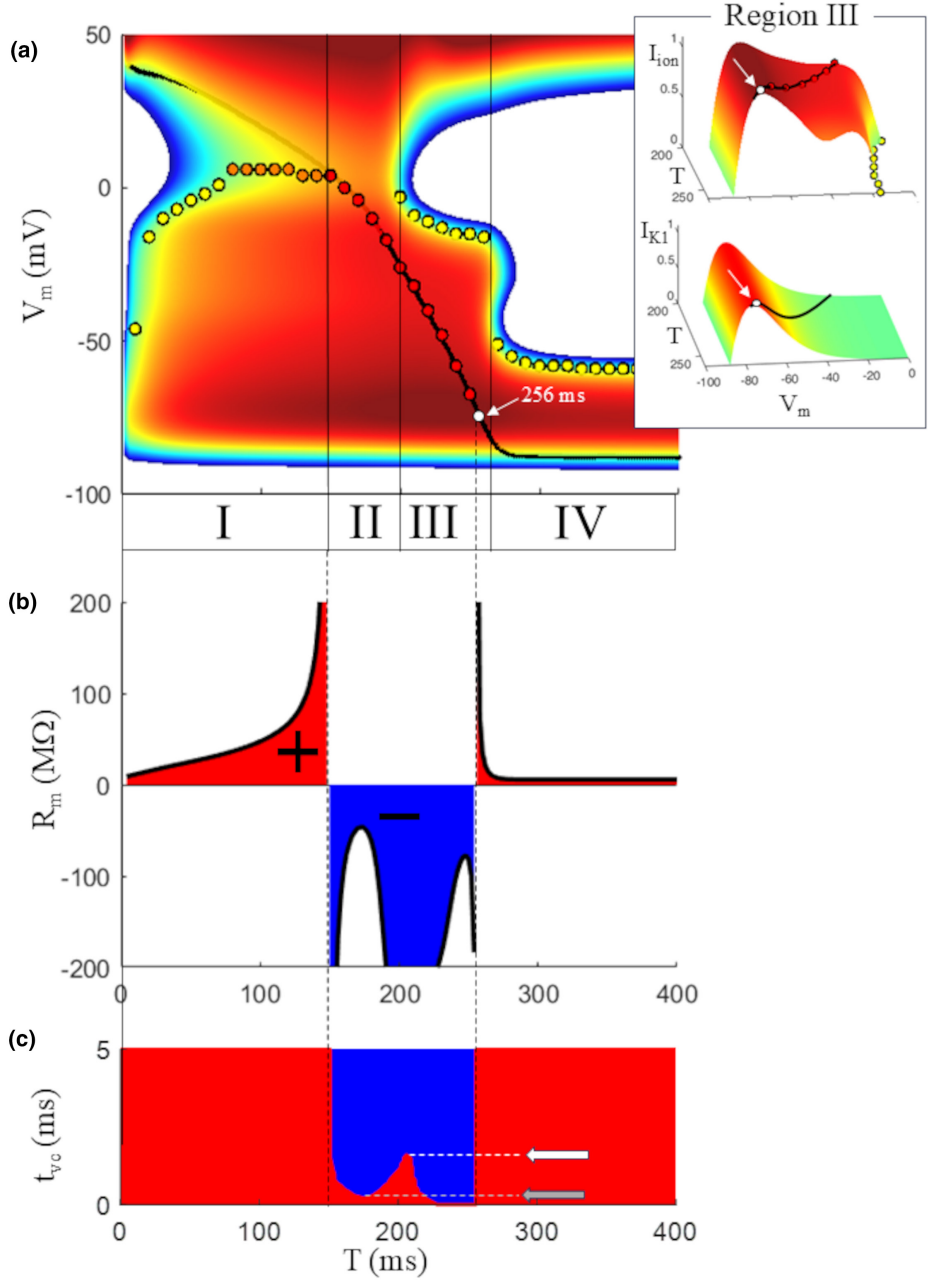
Summary of membrane resistance. (a) The IVT_5ms_ surface and thresholds already reported in Figure 9. The top panel of the inset shows region III from a different angle, the lower panel the corresponding IVT‐surface for *I*_K1_. White arrows and dots show the (I,V,T)‐state where IV‐curves pass from negative‐ to positive‐slope. (b) The slope of the surface was measured along IV planes at every 2 ms during the AP and provided the *R*_m_ values, reported as a black curve, with the area under the positive part of the curve in red, and that under the negative part in blue. The former coincide with regions I and IV defined in Figure 9, the latter with regions II and III. (c) Effect of varying *t*_vc_ (on the ordinate axis) on the distribution of positive‐ (red) and negative‐ (blue) sloping regions of the IVT surface.

Thus measured, *R*_m_ is positive during the initial plateau phase (T < 150 ms), when it progressively increases, and at rest (T > 260 ms), when it assumes a constant value (Figures 10 and 11b,c). In the same figure, the contribution of the main repolarization currents to the IVT‐surface is reported. For 150 ≤ T < 256 ms (Figure 11) the slope of the isochronal IV‐curves becomes negative and so does the value of *R*_m_, −114.8 MΩ at T = 180 ms and − 315.1 MΩ at T = 220 ms (out of scale in panel b). The negative sloping IV curve of *I*_Kr_ (red curves in panel d) seems to be the main responsible for this result. To note, the time windows identified on the base of threshold properties correspond to the sign of the slope of the surface at the (I,V,T)‐states occupied during the AP, and therefore with that of *R*_m_ (Figure 12, see also Movie S3). Note also that the “top of the *I*_ion_ hill”, almost entirely determined by *I*_K1_, is reached by the (I,V,T)‐state of the AP at time T = 256 ms (white dot in Figure 12a and in the inset with a detail of region III) where the slope of isochronal IV curves change their sign from negative (regions II and III, except for the last 4 ms) to positive (region IV). It is worth recalling that this same point marks the end of the region where repolarization is self‐regenerating and corresponds to the end of absolute refractory period as well, which points once again to the key role of *I*_K1_ (see bottom panel of the inset) in controlling late repolarization dynamics.

To emphasize differences (Figure 12b), the region under the positive *R*_m_ curve (bold black) is filled in red, in blue the negative. As noted above, all simulations shown in this study with the exceptions of those of Figures 2, 3, 4 and 6, have been made by setting *t*_vc_ = 5 ms. Figure 12c shows the effect of changing *t*_vc_ on the width of the negative *R*_m_ window (region II + III). The panel reports only the sign of *R*_m_ for any time T during AP repolarization and for surfaces simulated with *t*_vc_ values from 0.1 μs up to 10 ms (only interval 0–5 is reported in figure). Using *t*_vc_ from 10 down to 2 ms does not change the width of interval II + III. For *t*_vc_ smaller than 2 ms a positive sloping surface starts to appear in between negative regions (white arrow in panel c), until the width of the negative window collapses into a smaller T range for *t*_vc_ <0.2 ms (gray arrow). The slope of region II seems to be dominated by the negative slope of *I*_Kr_‐V curve, whereas that of region III by the fine interplay between negative sloping *I*_Kr_‐V, *I*_K1_‐V, and *I*_CaL_‐V curves (Figure 11d).

### 3.4 Safety of membrane repolarization

**FIGURE 13:**
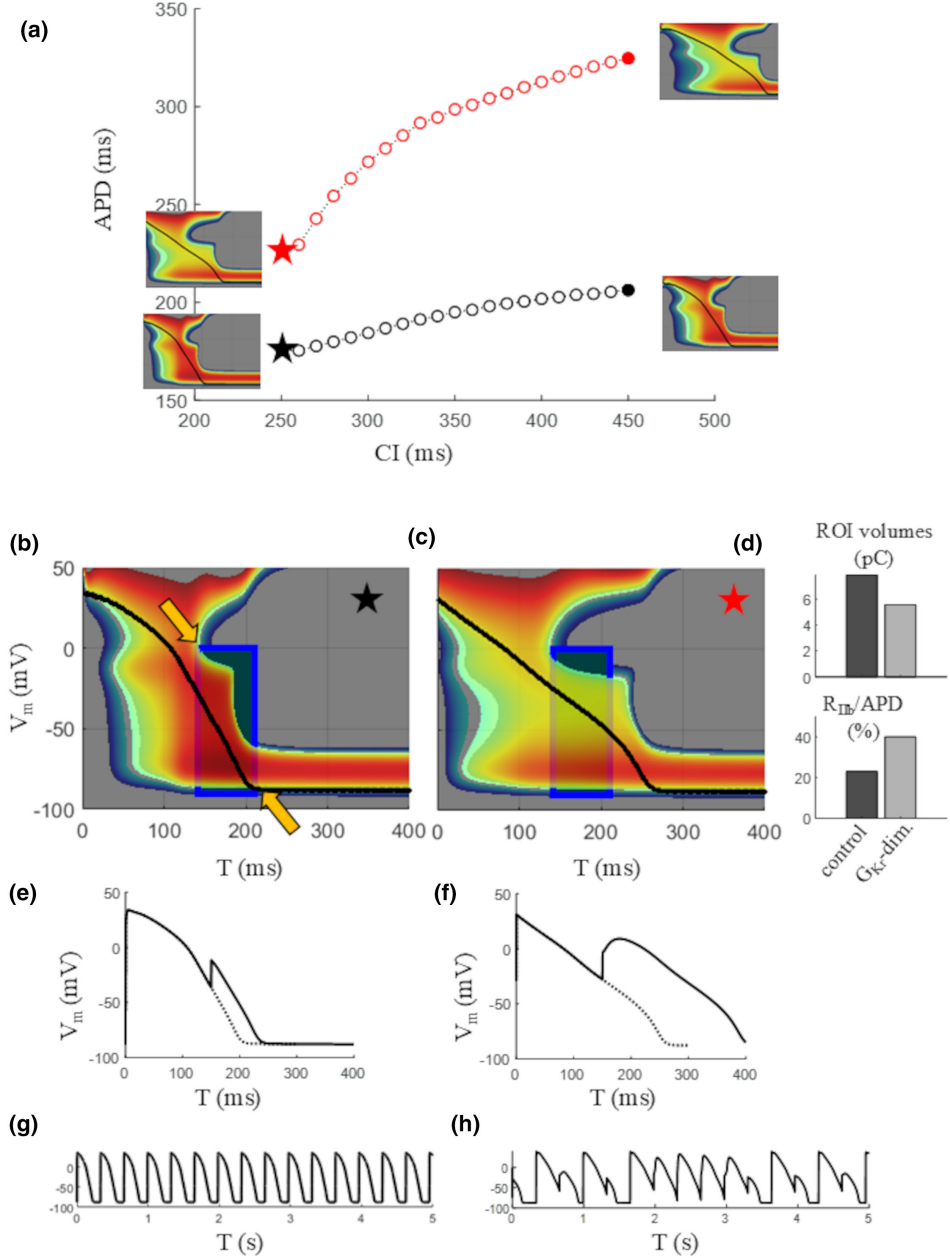
Safety of AP repolarization. (a) Standard APD restitution was measured in the ORd model paced at BCL = 350 ms in control conditions (empty black dots) and after reduction of *G*_Kr_ to 30% of its control value (empty red dots). The IVT_1ms_ surfaces of shortest (filled stars) and longest (filled circles) beats are reported as insets of the same panel. (b) A region of interest (ROI, blue rectangle) was established on the IVT surface of the shortest control APD, from the lower limit of region III to the 99% of APD repolarization. (c) An ROI of the same size was positioned on the IVT surface of the shortest *G*_Kr_‐diminished APD starting from the lower limit of region III. (d) Top histogram: The volume under each ROI divided by the voltage range provides the charge (in pC) available during that time in control (dark gray) and *G*_Kr_‐diminished (light gray) conditions. Bottom histogram: The ratio between duration of region III and APD is reported in the two cases. (e and f) An extra‐stimulus delivered at the same time within the ROI (T = 150 ms) in control (e) and *G*_Kr_‐diminished conditions (f) leads to a passive and active response respectively. (g) When pacing after the extra‐stimulus was kept at a constant CL = CI (coupling interval S2), normal and abnormal (alternant) repolarization was achieved.

Perhaps the most promising application of IVT‐surfaces is in quantifying the safety of AP repolarization, particularly in cases when AP trajectory undergoes pro‐arrhythmic events. The term safety for repolarization is related to mechanisms that guarantee a normal recovery of membrane resting potential when APD is abnormally increased, which can lead to arrhythmia risk (Varro et al., 2000). Pro‐arrhythmic conditions were simulated here by decreasing maximum conductance of *I*_Kr_ (*G*_Kr_) to 30% of its original value and monitored through electrical restitution (ER). Figure 13a (see also Movie S4) represents the ER curve as measured by simulating a standard S1‐S2 protocol (Zaniboni, 2023) on the ORd model driven at high pacing rate (BCL = 350 ms), in control (black) and in *G*_Kr_‐decreased condition (red). The IVT_1ms_ surfaces of the shortest and longest CIs are reported in the insets (labels are omitted for clarity). The IVT_1ms_ surfaces of the shortest beats are also reported in panel b (control) and c (*G*_Kr_‐ decreased). The volume underlying the control surface is measured in the region of interest (ROI) marked in blue, starting from the beginning of region III and ending when *V*_m_ reached 99% of its repolarization (orange arrows in panel b). The volume under the *G*_Kr_‐decreased surface is measured also from the beginning of region III and for the same amount of time (panel c). The two volumes, divided by the height of ROI (in mV), and assuming a *C*_m_ = 200 pF for a human cardiac myocyte (Polak & Fijorek, 2012), measure charge in pC, and are compared in the top histogram of panel d. The ratio between the width of region III (available threshold of *I*_CaL_) and APD is reported in the bottom panel d. The first parameter quantifies the amount of charge available to be translocated outside the membrane, either by ion currents or transporters, in a fixed time range during late repolarization, the second the relative duration of the time window when *I*_CaL_‐driven APs can be elicited. When in fact a 0.5 ms long extra‐stimulus was delivered at time T = 150 ms within the ROI in both the control and the *G*_Kr_‐ decreased cases (panels e and f), *V*_m_ recovered safely the AP trajectory and underwent an active response respectively. This, together with the obvious APD prolongation under 70% blockade of *I*_Kr_, contributes to explain why, when the shortest coupling interval is maintained constant in S1‐S2 protocol, the control condition retains normal repolarization whereas the modified one undergoes a complex APD alternans (panels g and h).

## 4 DISCUSSION

What I propose in this study is the use of quasi‐instantaneous current–voltage relationships for reconstructing a three‐dimensional representation of the cardiac ventricular AP that includes, together with time and membrane potential, also the total ion current available at any time during repolarization.

As opposed to steady‐state IV curves, which are commonly measured in voltage clamp in vivo experiments, quasi‐instantaneous IV curves are more difficult to measure in real patch clamped cardiac myocytes due to the need of repeatedly applying long conditioning trains and switching from current‐ to voltage‐clamp at given times during the AP of the same cell. Other authors have measured quasi‐instantaneous IV curves at few pre‐selected times during simulated AP repolarization, though sometimes introducing additional hypotheses on the activation probability of some ion channels and not discussing the key aspect of the length of voltage steps used to record instantaneous currents (Fink et al., 2006; Yang et al., 2015). Though I limit the present study to an in‐silico investigation, I note however that the protocol is potentially applicable, with some limitations, also in real cell experiments, as shown by previous measurements of quasi‐instantaneous IV curves during APs in patch clamped guinea pig ventricular myocytes (Zaniboni et al., 2000), and would greatly benefit studies investigating AP stability during plateau (Yang et al., 2015). I don't think the entire surface can be realistically reconstructed from real patch clamped cells with the detail of the present simulations, though selected regions of repolarization can be studied, for example the late phase, with the limited goal of estimating RR, or the plateau phase, for measuring thresholds of AONR.

*The choice of t*_vc_ A key point of the present study is the meaning and the limitations of the term quasi‐instantaneous for the IV curves measured during the AP. Would the object of investigation be the membrane of the giant axon of Loligo, then as *t*_vc_ gets shorter, the IV curve measured at every time T would tend to a straight line (Bezanilla et al., 1970), and the same would be for other preparations (Goldman & Morad, 1977). Ion currents in these early models are in fact instantaneously ohmic and their voltage‐dependence only develops in time following gating dynamics. In most of cardiac ventricular APs, and in their numerical reconstructions, though, there are ion currents with an instantaneous voltage‐dependent component (Grandi et al., 2010; Iyer et al., 2004; ten Tusscher & Panfilov, 2006) which develops further in time when *V*_m_ changes. Since it is so in the ORd model formulation in the case of, among others, *I*_K1_, *I*_CaL_, and *I*_Kr_ (O'Hara et al., 2011), straight IV curves are not expected as *t*_vc_ approaches zero. The question remains on how small *t*_vc_ should be set to better represent AP dynamics. The answer that it depends on what dynamical property one wants to emphasize is obvious but unsatisfying, and further quantitative considerations can be provided based on the obtained results. Simulations in Figures 5, 7, and 8 for example, concern the finding of thresholds that can be reached by injecting current across the membrane, which induce *V*_m_ displacements in it, with a delay proportional to its time constant τ (~ 2.5 ms in the ORd model). Thus, a *t*_vc_ value of twice the τ (5 ms) has been used when looking for thresholds during the AP. I note, on the other hand, (see Movies S1 and S2) that the morphology of the surface around the AP trajectory doesn't change substantially when *t*_vc_ varies from 0.1 μs up to 0.2 ms, and that, for *t*_vc_ >1 ms, thresholds for *I*_CaL_ and *I*_Na_ are clearly established. Moreover, the four regions of AP repolarization identified (Figures 9 and 12) on the basis of thresholds and *R*_m_ properties, do not change significantly over a wide *t*_vc_ range from 0.2 to 10 ms, as shown in the simulation of Figure 12c.

*Thresholds*. The validity of the *t*_vc_ = 5 ms choice when looking for thresholds induced by current injections is confirmed by comparison with other approaches to measure the same phenomena. In the case of current injection at resting potential (Figure 5) the voltage threshold for excitation was also measured by simulating 5 ms long depolarizing current injections of increasing amplitude until an AP was elicited (not shown). In these additional experiments *V*_m_ threshold was derived graphically by plotting the first derivative of current‐induced *V*_m_ deflection changed in sign (thus proportional to *I*_ion_) versus *V*_m_, and taking the *V*_m_ value where *I*_ion_ went from positive to negative. Thus measured, *V*_m_ threshold value was identical (−58 mV) to that provided by the intersection of IVT‐surface in Figure 5.

For what concerns thresholds for AONR, the term is frequently limited to the very initial phase of AP, dominated by *I*_Na_ and *I*_to_, where a progressive decrease of *I*_Na_ induced by TTX or other compounds leads to a condition when *V*_m_ either develops a full AP or abruptly repolarizes (Dong et al., 2006, 2011; Krishnan & Antzelevitch, 1991). The fact that not only the initial fast depolarization of the AP but also the early plateau phase can exhibit this all‐or‐none behavior has been observed in early studies on cardiac Purkinje fibers and papillary muscle preparations by Weidmann (1951), Cranefield et al. (1957), Vassalle (1966), in squid axon by Huxley (1959), and reviewed by Trenor et al. (2017) in a recent paper. These authors delivered anodal current pulses lasting few milliseconds during the AP and noticed, in certain phases of repolarization, two qualitative different responses depending on the strength of the injected current. For current pulses of smaller strength, repolarization recovered the unperturbed AP trajectory after the release of the pulse, whereas, as the strength increased above a certain threshold, the membrane potential returned abruptly to its resting value, thus interrupting the AP course. To better characterize the phenomenon, Beeler and Reuter used a different protocol in their first model of mammalian ventricular AP (Beeler & Reuter, 1977). At different times during AP repolarization, they switched from current‐ to voltage‐clamp mode and clamped the membrane at progressively more polarized 5 ms long *V*_m_ steps. After the clamp release, membrane potential showed the dual behavior described above, making it possible to identify the *V*_m_ threshold (highlighted white dots in Figures 7e and 8e) where this transition occurred. Also in this case (see Figures 7, 8, 9), threshold values found from IVT‐surface inspection were identical to those measured by applying the Beeler and Reuter protocol. A common feature of resumed *V*_m_ traces (Figures 7d and 8d) is a clear inversion of the direction of growth of their averaged first time derivative (average rate of de/re‐polarization) when starting from more depolarized potentials (green arrows in Figures 7e and 8e). This allows identifying transition to SRR, either through thresholds (yellow dots in Figures 7, 9, and 12) or not (orange dots in the same figures), also in cases where the inspection of *V*_m_ traces of Figure 7d would be more difficult and largely arbitrary. Moreover, either *V*_m_ thresholds for excitation or for AONR were confirmed by anodal and cathodal current injections during the AP (Figure 9). The threshold for AONR has been linked to the safety of repolarization in studies of proarrhythmic risk associated with pharmacological treatments, where, in addition, RR plays a role (Fink et al., 2006; Trenor et al., 2017). IVT surfaces allow to extend evaluation of safety of repolarization independently from the AONR phenomenon and, as it will be discussed below, provide a quantitative estimate of RR.

*Membrane resistance during AP*. The measure of membrane resistance during the cardiac AP has interested physiologists since the early studies of Weidmann (1951) for its role in modulating repolarization, particularly under electrotonic interaction (Spitzer et al., 2006; Zaniboni et al., 2000), and has been exhaustively reviewed by Trenor and colleagues in a recent paper (Trenor et al., 2017). The IVT representation of AP makes *R*_m_ evaluation intuitive and readily accessible by inspection of the corresponding surface (Figures 10 and 11). I have used a similar approach in previous studies (Zaniboni, 2011, 2012a, 2012b; Zaniboni et al., 2010), though, as mentioned above, focusing on *I*_m_‐ rather than *I*_ion_‐*V*_m_ representations, and not considering the impact of varying *t*_vc_ on results.

IVT‐surfaces allow studying in detail the AP phase during late repolarization where surface is relatively flat, thus associated with very high *R*_m_. This phase (regions II and III in Figures 9 and 12) is characterized by the competing interplay of the negative slopes of *I*_Kr_‐V and *I*_CaL_‐V curves (left and middle panels of Figure 11d), which lead to a rather flat total IV curve and to a tiny overall net outward current (left and middle panels of Figure 11c). The *R*_m_ increase during ventricular AP plateau and its return to the range of few MΩ at diastolic potentials (Figure 12b) has been described previously (Spitzer et al., 2006; Weidmann, 1951; Zaniboni, 2011, 2012b; Zaniboni et al., 2000, 2010). Some have reported much higher values (Kaur et al., 2014). The quasi‐ohmic nature of *R*_m_ during plateau phase and diastole is also made manifest in the near symmetrical *I*_ion_ deflection in response to symmetrical ΔV_m_s (Figure 3d). I have discussed in detail the diastolic deviation of *R*_m_ from ohmicity in a previous paper (Zaniboni et al., 2005). On the other hand, to date, the critical behavior of *R*_m_ during the late phase of repolarization has received less attention. A negative sloping IV curve in electronics is associated with circuit elements that do not absorb but generate power (Sarafian, 2021), and the negative values of *R*_m_ during late AP trajectory suggest, in principle, that the membrane acts as an active, self‐regenerating, source of repolarization during this phase. This is a very tempting hypothesis which would shed light on the still puzzling mechanism underlying the propagation of ventricular repolarization (Himeno et al., 2023; Noble & Hall, 1963). The present study, on the other hand, does not include simulations of electrotonic AP propagation, and considerations on source‐sink dynamics remain only speculative.

*Current available and safety of AP repolarization*. The main advantage provided by IVT representations is to make available at once, together with the AP, key dynamical properties of repolarization independently from the knowledge of underlying ion currents. The three‐dimensional representation provides in fact, together with time T and *V*_m_ of the AP, the *I*_ion_ available at any *V*_m_ at any given time T. It displays, in other words, at any time T, the *I*_ion_ that would flow when *V*_m_ would quasi‐instantly be displaced from the AP trajectory. In this sense is referred throughout this article as available current, and it is linked with safety and reserve of repolarization. Quantifying RR can better explain, for instance, the increased pro‐arrhythmic risk associated with class III anti‐arrhythmic drugs (Zaniboni, 2023). When *G*_Kr_ reduction was simulated, in fact, in addition to the expected APD prolongation and to the arrhythmogenic increase of ER slope up to 1.34 predicted according to the restitution hypothesis (Ideker et al., 2002; Zaniboni, 2023), the inspection of IVT‐surface could add further light into the increased risk of arrhythmia development. IVT‐surface shows, in this case, the dramatic decrease in outward charge available in the late repolarization phase (Figure 13d, top panel), as well as the increase in the fraction of AP time (region III) during which a threshold for calcium‐triggered AP is available. These two facts make it possible for an anticipated extra‐stimulus falling in this phase to trigger a calcium‐driven AP in the *G*_Kr_‐diminished condition, leading in turn to APD alternans (Figure 13h), a well‐recognized trigger of ventricular arrhythmias (Rosenbaum et al., 1994). The volume underlying the IVT‐surface multiplied by the voltage range over which it is measured (height of the blue ROI in panels c and d) describes in fact the charge available to be mobilized in outward direction by either ion channels or membrane transporters in the corresponding time window (width of the ROI), charge that can contribute to make final repolarization safer when AP trajectory is perturbed, for example by an ectopic beat. Though RR is recognized as a key factor in protecting ventricular AP from abnormal repolarization (Fink et al., 2006; Roden, 2008; Roden & Abraham, 2011), this parameter is frequently used only to qualitatively indicate a redundance of repolarizing currents during the AP (Roden, 2008; Roden & Abraham, 2011; Wu & Anderson, 2002).

Quantitative estimates of RR have been provided by other studies, though with a quite different meaning. On one hand, multiple perturbations of ion currents have been considered all together in their statistical effect on one significant marker of membrane AP repolarization, like APD (Sarkar & Sobie, 2011), on the other hand new markers have been proposed, like the charge carried by the total ion current at the end of repolarization (Dutta et al., 2017) or the minimum constant current preventing normal repolarization (Gaur et al., 2020), more effective than APD in evaluating repolarization robustness against perturbations. In all cases RR was estimated for a given AP waveform, that is, as a metric in a two‐dimensional space.

The method I'm proposing here for quantifying RR in the (I,V,T)‐space has the advantage of associating this property with a physical entity measurable in that space, i.e. the amount of positive/negative charge available to cross the membrane in outward/inward direction and guarantee safety of repolarization during AP trajectory or when trajectory is perturbed. When considering RR, in other words, the key factor is not how much current is flowing during late AP repolarization, but rather the amount of current, or the amount of charge, that is available when *V*_m_ deviates from the original AP trajectory, which is indeed measured by the volume under the IVT‐surface at that time (Figure 13b–d). This way of looking at RR as a given amount of charge available, indeed measurable in pC, makes its estimate more intuitive and univocal when compared with previously described metrics.

## 5 CONCLUSIONS

What I present here are only few examples, taken from the basic ventricular AP physiology, where IVT‐surfaces representation of the AP can draw new light into and unify our understanding of excitation and repolarization dynamics. Among the others, the most relevant finding is the possibility of quantifying RR. Based on preliminary experiments with the same approach, I believe that this method can tell much more on general mechanisms underlying AP modification during pharmacological and electrotonic modulation of cardiac electrical activity, where a unifying dynamical picture is required to make sense of the complex interactions underlying physio‐pathological transitions in the ventricular function. As previous attempts have shown, nothing prevents from applying the same approach on in vivo patch clamped cardiac myocytes, though a mathematical description of the phenomenon was due before undertaking a not so easy experimental task. Furthermore, given the weight that computational electrophysiology has assumed in paralleling in vivo research, the three‐dimensional representation of ventricular AP presented here is relevant per se, as an additional tool for studying AP repolarization and for targeting its key dynamical features.

## FUNDING INFORMATION

This work has benefited from the equipment and framework of the COMP‐HUB Initiative, funded by the ‘Departments of Excellence’ program of the Italian Ministry for Education, University and Research (MIUR, 2018‐2022).

## ETHICS STATEMENT

None.

## Supporting information


Movie S1.

Movie S2.

Movie S3.

Movie S4:

## Data Availability

The data that support the findings of this study are available from the corresponding author upon reasonable request.
